# Prevalence and risk factors for anaemia during pregnancy in Sylhet district of Bangladesh: a cohort study

**DOI:** 10.7189/jogh.16.04016

**Published:** 2026-02-20

**Authors:** Tarik Hasan, Rasheda Khanam, Nabidul Haque Chowdhury, Diwakar Mohan, Salahuddin Ahmed, Sayedur Rahman, Md Shafiqul Islam, Arunangshu Dutta Roy, Debarati Ghosh, Md Biplob Hosen, Rakib Ullah Kuddusi, Rubhana Raqib, Sachiyo Yoshida, Sunil Sazawal, Fyezah Jehan, Abdullah H Baqui

**Affiliations:** 1 Projahnmo Research Foundation, Dhaka, Bangladesh; 2 Department of International Health, Johns Hopkins Bloomberg School of Public Health, Baltimore, Maryland, USA; 3 International Center for Diarrhoeal Disease Research, Bangladesh, Mohakhali, Dhaka, Bangladesh; 4 Department for Maternal, Child, Adolescents and Ageing Health, World Health Organization, Geneva, Switzerland; 5 Center for Public Health Kinetics, New Delhi, India; 6 Department of Pediatrics and Child Health, The Aga Khan University, Karachi, Pakistan

## Abstract

**Background:**

Anaemia during pregnancy poses a major public health problem globally, with reported prevalences ranging from approximately 5.2% to 65.7%. A significant portion of this burden is borne by low- and middle-income countries. We aimed to determine the prevalence of anaemia during pregnancy and identify the factors associated with anaemia in the third trimester of pregnancy in a cohort of women in the rural Sylhet district, Bangladesh.

**Methods:**

We enrolled 3000 pregnant women between 8 and 19 weeks of gestational age (GA). Trained community health workers collected data on their sociodemographic, obstetric, nutritional, dietary, anthropometric, and household characteristics. Blood samples were collected at baseline (<20 weeks of gestation) and at a follow-up visit between 24–36 weeks of GA to measure haemoglobin (Hb) concentrations. We classified them according to their anaemia status into no anaemia (Hb ≥11g/dl), mild (10 to <11 g/dl), and moderate to severe (<10g/dl) groups. We then used unadjusted and adjusted multinomial logistic regression models to calculate the relative risk ratios (RRR) and 95% confidence intervals (CIs) of potential risk factors for mild or moderate to severe anaemia in the third trimester of pregnancy.

**Results:**

Of the 2679 women tested at enrolment, 1010 (38%) were anaemic, 634 (24%) had mild anaemia, and 376 (14%) had moderate to severe anaemia. At the third-trimester follow-up, 1950 (79%) of 2473 women were anaemic; 739 (30%) had mild anaemia, and 1211 (49%) had moderate to severe anaemia. Women with baseline mild anaemia had about a five-fold higher risk (RRR = 4.84; 95% CI = 3.64–6.46) and those with moderate to severe anaemia about an 11-fold higher risk (RRR = 10.78; 95% CI = 6.69–17.35) of having moderate to severe anaemia in the third trimester. Iron supplementation during pregnancy (RRR = 0.75; 95% CI = 0.60–0.93) and drinking tubewell water (RRR = 0.76; 95% CI = 0.60–0.96) were significantly associated with a lower risk of moderate to severe anaemia in the third trimester.

**Conclusions:**

We documented a high rate of anaemia in our sample, particularly during the third trimester of pregnancy, underscoring an urgent need for interventions to improve maternal and child health outcomes, such as improved nutrition education, expanded access to and adherence with iron supplementation, and enhanced antenatal care.

Anaemia affects about 1.62 billion people worldwide annually [1,2]. While it occurs in almost all age groups, its prevalence is highest among pregnant women, with an estimated 56 million being anaemic each year [3–5]. The prevalence is much higher in low- and middle-income countries (LMICs), at ~56%, than in developed countries (18%) [6–8].

The presence of anaemia during pregnancy can lead to adverse effects for both the mother and the foetus, increasing the risk of miscarriage, stillbirth, prematurity, and low birth weight [5,9–11]. Several studies from South Asia have found a high prevalence of anaemia among pregnant women (62–91%) [12–14], as did research from Bangladesh, specifically (37–64%) [4,7,15–17].

According to the Centers for Disease Control and Prevention (CDC) and the World Health Organization (WHO), pregnant women with haemoglobin (Hb) levels of 11 g/dl or higher are considered normal (*i.e.* to have no anaemia), and those with Hb <11 g/dl are considered anaemic [6–9]. The severity of the condition is further classified by the WHO as mild (10.0–10.9 g/dl), moderate (7.0–9.9 g/dl), and severe (<7 g/dl) [4,6,8,9]. Several factors, including sociodemographic characteristics such as age, education, income, residence, and clinical conditions (*e.g.* history of excess menstrual bleeding, short interpregnancy interval, and parity), influence Hb concentrations [11,18,19], while dietary and nutritional practices have been proven as risk factors for anaemia [6,20].

Iron deficiency is the most common cause of anaemia, which affects up to 50% of pregnant women globally [21,22]. Despite the high prevalence of anaemia among pregnant women in Bangladesh, we lack comprehensive data on related risk factors, including dietary practices during pregnancy. Using prospectively collected data from a cohort of pregnant women from the rural Sylhet district of Bangladesh, we sought to estimate the prevalence of anaemia at two time points in pregnancy at 8–19 weeks of gestation (baseline) and in the third trimester at 24–36 weeks of gestation (follow-up), and to identify the risk factors by severity during the third trimester of pregnancy.

## METHODS

### Study design, participants, and settings

We used data from the Alliance for Maternal and Newborn Health Improvements pregnancy cohort, the methodology of which has been detailed elsewhere [23]. Briefly, we recruited 3000 pregnant women from Zakiganj and Kanaighat rural subdistricts in Sylhet District in northeastern Bangladesh. Between 2014 and 2018, trained community health workers (CHWs) made bimonthly home visits to identify pregnant women, confirming pregnancies using a strip-based pregnancy test. After obtaining informed consent, women were invited to have an ultrasound to determine the duration of gestation; those with pregnancies of 8–19 weeks were enrolled in the study. Using piloted questionnaires, the CHWs collected women’s sociodemographic, obstetric, clinical, nutritional, dietary, and household data. They also collected data on the weight and height of women before 20 weeks of gestation using standard methods.

### Blood collection and measurement of Hb concentrations

Blood samples were collected at two different time points: at baseline (8–19 weeks of gestation) and at follow-up in the third trimester of pregnancy (24–36 weeks). A trained paramedic collected blood samples into an EDTA tube using a disposable butterfly needle from the mother’s antecubital vein, following all aseptic precautions. Trained laboratory technicians assessed Hb concentration after they extracted blood from the collection tube immediately using the auto-calibrated Hemocue Hb 201 analyser (Hemocue, Inc., Ängelholm, Sweden) and a specially-designed microcuvette, which is widely used in field settings due to its portability and accuracy. Quality control was done weekly and with each new batch of microcuvettes using both high and low control solutions to ensure measurement reliability.

We categorized the reference values of Hb according to the WHO criteria as normal (11g/dl or higher) or anaemic (<11 g/dl), and the severity of the anaemia as mild (10.0–10.9 g/dl), moderate (7.0–9.9 g/dl), or severe (<7.0 g/dl) [7,10,11]. We combined the latter two categories, as we had too few severe cases to ensure sufficient statistical precision and power. CHWs, paramedics, and laboratory technologists strictly followed standard operating procedures (SOPs) during data and specimen collection and Hb measurement to ensure quality: they underwent regular retraining to minimise errors in data collection or specimen processing, and were monitored by supervisors to ensure data quality.

### Measurements

The primary outcome was anaemia during the third trimester of pregnancy (24–36 weeks of gestation). The main explanatory variable was anaemia status at baseline. Maternal factors (maternal age, education, depressive symptoms using patient health questionnaire – 9 (PHQ-9)); dietary data (consumption of meat, milk, lentils, fish, green leafy vegetables, and fruits); iron intake; maternal weight and height; husband’s education; family size; household wealth status; and drinking water source acted as covariates in the analysis. We categorised maternal age as 15–19, 20–29, and ≥30 years; woman’s and husband’s education into 0–5 years and ≥6 years of schooling; and maternal depression as a PHQ-9 score of <10 and ≥10. We calculated the body mass index (BMI) using maternal weight and height data and classified it into underweight (<18.5 kg/m^2^), normal (18.5–24.9 kg/m^2^), and overweight/obese (25 kg/m^2^ or more). Using principal component analysis, we created household wealth scores using data on housing materials and household assets and grouped them into household wealth tertiles.

We assessed dietary intake using a seven-day food frequency recall questionnaire, which had been administered by trained interviewers. This method has been previously applied in similar rural Bangladesh settings, where a seven-day semiquantitative Food Frequency Questionnaire (FFQ) demonstrated moderate to good validity compared with multiple 24-hour recalls (Lin’s concordance coefficients = 0.21–0.90) [24]. The study also showed good correlations between the seven-day FFQ and the average of two 24-hour dietary recalls (*ρ* = 0.46–0.85; *P* < 0.001) with the key micronutrients studied, including iron, zinc, calcium, and vitamin [24]. For this analysis, we categorised food consumption frequency as 0–1 or ≥2 times per week based on the distribution of reported intakes in the study population, reflecting meaningful differences in habitual consumption, rather than precise quantitative estimates.

### Statistical methods

We used bivariate analyses to examine the relationship between independent and outcome variables. given that the outcome variable (anaemia in the third trimester of pregnancy) and the primary predictor variable (anaemia at enrolment) were categorical with three levels (none, mild, and moderate to severe), we used multinomial logistic regression to investigate the effect of baseline anaemia status on the anaemia status in the third trimester of pregnancy. This approach allowed for simultaneous estimation of the probability of being in one anaemia category relative to a reference category.

### Multinomial model specification

Let *Y* denote the outcome variable (anaemia status at 24–36 weeks) with *k* categories (*k* = 3), and *X* represents the predictor variable (anaemia status at baseline). The multinomial logistic model estimates the probability of being in each non-reference category of *Y* relative to the reference category (no anaemia), conditional on *X*. For each category *j* of the outcome (where *j* = 1,2) corresponding to mild anaemia and moderate to severe anaemia), the log-odds of *Y* = *j* relative to the reference category *Y* = 0 (no anaemia) are modelled as:



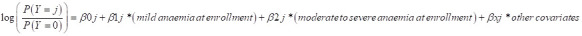



Where *P*(*Y* = *j*) represents the probability of the outcome being in category *jj*; *P*(*Y* = 0) is the probability of being in the reference category (no anaemia); *β*0*j* is the intercept for category *j*; *β*1*j* is the coefficient for those with ‘mild anaemia at enrolment’, representing the log-odds change of being in category *j* relative to the reference category; *β*2*j* is the coefficient for those with ‘moderate to severe anaemia at enrolment’, representing the log-odds change of being in category *j* relative to the reference category.

The designation of the ‘no anaemia’ category as the reference for both the predictor and the outcome variables allows for a clear interpretation of the odds ratios, quantifying the relative risk of developing mild or moderate to severe anaemia at 24–36 weeks compared to remaining non-anaemic.

We implemented the model using Stata, version 17 (StataCorp LLC, College Station, Texas, USA) by utilising the ‘mlogit’ function, deriving the relative risk ratios (RRRs) with corresponding 95% confidence intervals (CIs) for each anaemia category relative to the reference. These risk ratios quantify the likelihood of progression or persistence of anaemia across the pregnancy period based on initial anaemia status. We evaluated statistical significance at the *α* = 0.2 level to select variables for the multivariable model. We also generated the predicted probabilities for the transition to the different states of anaemia and plotted them with 95% CI. The proportion of missing observations across covariates was minimal, at ~ 5%. We used the hot-deck imputation method to address missing data.

## RESULTS

**Figure 1:**
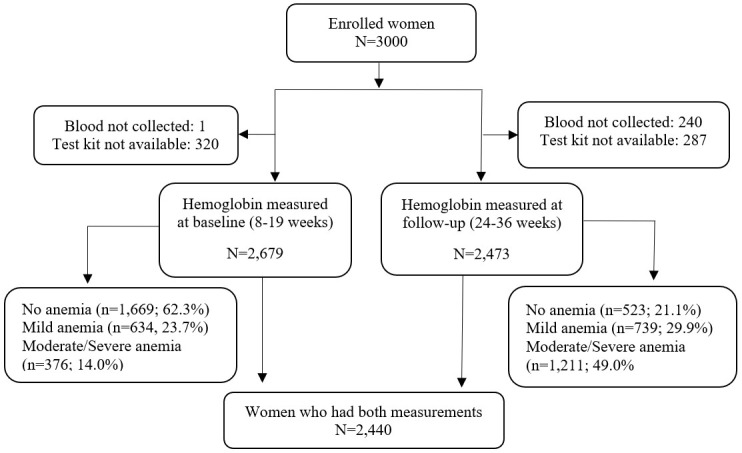
Study flowchart.

Out of 3000 enrolled women, Hb was measured at baseline for 2679 and at follow-up for 2473 women, whereby 2440 women had Hb measured at both time points (**Figure 1**). At baseline, approximately 38% of the women were anaemic, with about 24% having mild anaemia and 14% having moderate to severe anaemia. The prevalence of anaemia at follow up was 79%, with about 30% having mild anaemia and 49% having moderate to severe anaemia. Reasons for missing data include blood not being collected (one at baseline, 240 at follow-up) and unavailability of test kits (320 at baseline, 287 at follow-up).

Maternal anaemia at baseline, maternal age, maternal BMI, receiving iron supplements, drinking tube-well water, consumption of green leafy vegetables, consumption of any fish, and consumption of meat were associated with maternal anaemia categories at follow-up (**Table 1**).

**Table 1:** Maternal and household characteristics by anaemia status, measured between 24–36 weeks of GA, n (%)

	Anaemia at 24–36 weeks of gestation				
	**No anaemia**	**Mild anaemia (10.0–10.9 g/dl)**	**Moderate to severe anaemia (<9.9 g/dl)**	**Total**	***P*-value**
**Baseline anaemia (8–19 weeks of gestation)**					0.000
None	428 (28.3)	557 (36.9)	525 (34.8)	1,510	
Mild	70 (12)	112 (19.1)	403 (68.9)	585	
Moderate severe	20 (5.8)	58 (16.8)	267 (77.4)	345	
**Mother’s age**					0.069
15–19 y	101 (19.6)	143 (27.8)	271 (52.6)	515	
20–29 y	369 (22.6)	492 (30.1)	775 (47.4)	1636	
≥30 y	48 (16.6)	92 (31.8)	149 (51.6)	289	
**Maternal education**					0.236
0–5 y	213 (20.6)	295 (28.5)	528 (51)	1036	
≥6 y	305 (21.7)	432 (30.8)	667 (47.5)	1404	
**Husband education**					0.505
0–5 y	329 (20.5)	484 (30.2)	788 (49.2)	1601	
≥6 y	189 (22.5)	243 (29)	407 (48.5)	839	
**Wealth status**					0.445
Low	160 (19.2)	261 (31.4)	411 (49.4)	832	
Middle	181 (22.2)	241 (29.5)	394 (48.3)	816	
High	177 (22.3)	225 (28.4)	390 (49.2)	792	
**Family size**					0.997
≤4	183 (21.3)	256 (29.8)	420 (48.9)	859	
≥5	335 (21.2)	471 (29.8)	775 (49)	1581	
**Depressive symptoms during pregnancy**					0.501
PHQ-9 < 10	443 (21.5)	619 (30)	999 (48.5)	2061	
PHQ-9 ≥ 10)	75 (19.8)	108 (28.5)	196 (51.7)	379	
**Source of drinking water**					0.008
Others	172 (19.3)	247 (27.7)	474 (53.1)	893	
Tubewell	346 (22.4)	480 (31)	721 (46.6)	1547	
**Iron intake**					0.056
No	252 (21)	334 (27.8)	616 (51.2)	1202	
Yes	266 (21.5)	393 (31.7)	579 (46.8)	1238	
**Maternal BMI**					<0.001
Underweight (<18.5)	145 (18.3)	241 (30.4)	407 (51.3)	793	
Normal weight (18.5–24.9)	324 (21.7)	432 (29)	734 (49.3)	1490	
Overweight (≥25)	49 (31.2)	54 (34.4)	54 (34.4)	157	
**Any meat consumption in 24–28 weeks GA**					0.087
Never/once a week	300 (19.8)	458 (30.2)	757 (50)	1,515	
≥2 times a week	218 (23.6)	269 (29.1)	438 (47.4)	925	
**Milk**					0.551
Never/once a week	470 (21)	672 (30.1)	1093 (48.9)	2235	
≥2 times a week	48 (23.4)	55 (26.8)	102 (49.8)	205	
**Any fish consumption in 24–28 weeks GA**					0.151
Never/once a week	51 (20.8)	61 (24.9)	133 (54.3)	245	
≥2 times a week	467 (21.3)	666 (30.3)	1062 (48.4)	2,195	
**Lentils consumption in 24–28 weeks GA**					0.602
Never/once a week	317 (21.2)	456 (30.5)	722 (48.3)	1495	
≥2 times a week	201 (21.3)	271 (28.7)	473 (50.1)	945	
**Green leafy vegetables consumption in 24–28 weeks GA**					0.043
Never/once a week	166 (18.9)	256 (29.1)	457 (52)	879	
≥2 times a week	352 (22.5)	471 (30.2)	738 (47.3)	1,561	
**Any fruit consumption in 24–28 weeks GA**					0.539
Never/once a week	372 (20.7)	535 (29.8)	889 (49.5)	1796	
≥2 times a week	146 (22.7)	192 (29.8)	306 (47.5)	644	

BMI – body mass index, GA – gestational age, PHQ-9 – Patient Health Questionnaire – 9

Moderate to severe anaemia at baseline significantly increased the risk of mild anaemia at 24–36 weeks GA. A higher risk of moderate to severe anaemia was associated with mild and moderate to severe anaemia at baseline among older women and among women who consume no meat and green leafy vegetables or do so once a week. Women drinking tube well water and overweight women had a significantly lower risk for moderate to severe anaemia at 24–36 weeks of GA. Depressive symptoms during pregnancy (PHQ-9 ≥10) were not significantly associated with anaemia at 24–36 weeks of gestation (**Table 2**).

**Table 2:** Unadjusted association of risk factors for anaemia by anaemia categories, measured at 24–36 weeks of gestation

	Mild anaemia, RRR (95% CI)	Moderate to severe anaemia, RRR (95% CI)
**Baseline anaemia (8–19 weeks of gestation)**		
None	ref	ref
Mild	1.23 (0.89–1.70)	4.69 (3.53–6.24)
Moderate to severe	2.23 (1.32–3.76)	10.88 (6.79–17.45)
**Mother's age**		
15–19 y	1.06 (0.80–1.42)	1.28 (0.99–1.66)
20–29 y	ref	ref
≥30 y	1.44 (0.99–2.09)	1.48 (1.04–2.09)
**Maternal education**		
0–5 y	0.98 (0.78–1.23)	1.13 (0.92–1.40)
≥6 y	ref	ref
**Husband education**		
0–5 y	1.14 (0.90–1.45)	1.11 (0.90–1.38)
≥6 y	ref	ref
**Wealth status**		
Low	1.23 (0.93–1.61)	1.18 (0.92–1.52)
Middle	ref	ref
High	0.95 (0.72–1.26)	1.01 (0.79–1.30)
**Family size**		
≤4	ref	ref
≥5	1.01 (0.79–1.27)	1.01 (0.81–1.25)
**Depressive symptoms during pregnancy**		
PHQ-9 < 10	ref	ref
PHQ-9 ≥ 10	1.03 (0.75–1.42)	1.16 (0.87–1.55)
**Source of drinking water**		
Others	ref	ref
Tubewell	0.97 (0.76–1.23)	0.76 (0.61–0.94)
**Iron intake**		
No	ref	ref
Yes	1.11 (0.89–1.40)	0.89 (0.72–1.09)
**Maternal BMI**		
Underweight (<18.5)	1.25 (0.97–1.60)	1.24 (0.98–1.56)
Normal (18.5–24.9)	ref	ref
Overweight (≥25)	0.83 (0.55–1.25)	0.49 (0.32–0.73)
**Any meat consumption in 24–28 weeks GA**		
≥2 times a week	ref	ref
Never/once a week	1.24 (0.98–1.56)	1.26 (1.02–1.55)
**Milk consumption in 24–28 weeks GA**		
≥2 times a week	ref	ref
Never/once a week	1.25 (0.83–1.87)	1.09 (0.76–1.57)
**Any fish consumption in 24–28 weeks GA**		
≥2 times a week	ref	ref
Never/once a week	0.84 (0.57–1.24)	1.15 (0.82–1.61)
**Lentils consumption in 24–28 weeks GA**		
≥2 times a week	ref	ref
Never/once a week	1.07 (0.85–1.35)	0.97 (0.78–1.20)
**Green leafy vegetables consumption in 24–28 weeks GA**		
≥2 times a week	ref	ref
Never/once a week	1.15 (0.91–1.46)	1.31 (1.06–1.63)
**Any fruits consumption in 24–28 weeks GA**		
≥2 times a week	ref	ref
Never/once a week	1.09 (0.85–1.41)	1.14 (0.90–1.44)

CI – confidence interval, GA – gestational age, ref – reference, PHQ-9 – Patient Health Questionnaire – 9, RRR – relative risk ratios

Pregnant women with mild anaemia at enrolment were at an increased risk of having moderate to severe anaemia (RRR = 4.84; 95% CI = 3.64–6.46) later in pregnancy. Those with moderate to severe anaemia at baseline had a higher risk of having mild anaemia (RRR = 2.11; 95% CI = 1.25–3.58) and moderate to severe anaemia (RRR = 10.78; 95% CI = 6.69–17.35) later in pregnancy. Overweight women (RRR = 0.57; 95% CI = 0.37–0.89) and those who used tubewells as a drinking water source (RRR = 0.76; 95% CI = 0.60–0.96) or reported intake of iron supplements (RRR = 0.75; 95% CI = 0.60–0.93) had decreased risk while those who ate meat no or once a week were at a higher risk of developing moderate to severe anaemia (RRR = 1.27; 95% CI = 1.01–1.60) later in pregnancy (**Table 3**).

**Table 3:** Risk factors for anaemia by anaemia categories at 24–36 weeks of gestation, after adjusting for covariates

	Mild anaemia *vs*. no anaemia, RRR (95% CI)	Moderate to severe anaemia *vs*. no anaemia, RRR (95% CI)
**Baseline anaemia status**		
None	ref	ref
Mild	1.21 (0.88–1.68)	4.84 (3.64–6.46)
Moderate to severe	2.11 (1.25–3.58)	10.78 (6.69–17.35)
**Mother's age**		
15–19 y	1.05 (0.78–1.40)	1.20 (0.91–1.58)
20–29 y	ref	ref
≥30 y	1.45 (1.00–2.12)	1.42 (0.98–2.06)
**BMI**		
Underweight (<18.5)	1.24 (0.97–1.60)	1.15 (0.90–1.47)
Normal	ref	ref
Overweight (≥25)	0.85 (0.56–1.28)	0.57 (0.37–0.89)
**Drinking water**		
Others	ref	ref
Tubewell	0.99 (0.77–1.26)	0.76 (0.60–0.96)
**Iron intake**		
No	ref	ref
Yes	1.09 (0.87–1.37)	0.75 (0.60–0.93)
**Any meat consumption in 24–28 weeks GA**		
≥2 times a week	ref	ref
Never/once a week	1.20 (0.95–1.52)	1.27 (1.01–1.60)
**Any fish consumption in 24–28 weeks GA**		
≥2 times a week	ref	ref
Never/once a week	0.78 (0.51–1.18)	1.15 (0.78–1.69)
**Any vegetable consumption in 24–28 weeks GA**		
≥2 times a week	ref	ref
Never/once a week	1.17 (0.91–1.51)	1.17 (0.91–1.50)

BMI – body mass index, CI – confidence interval, GA – gestational age, PHQ-9 – Patient Health Questionnaire – 9, ref – reference, RRR – relative risk ratios

**Figure 2:**
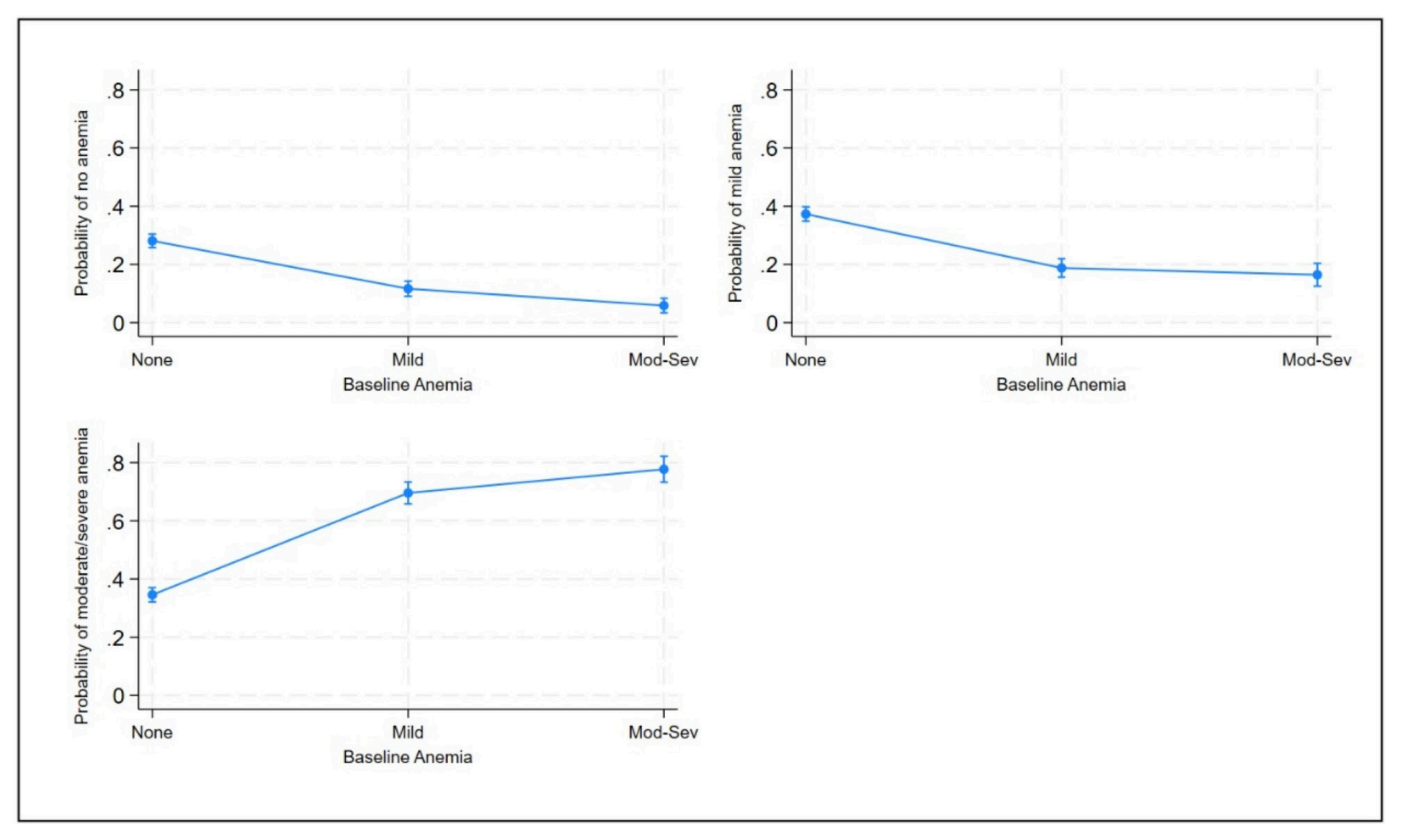
Predicted probabilities of categories at 24–36 weeks of gestation.

Women who did not have anaemia at baseline had probabilities of 11.7% and 5.9% to develop mild or moderate to severe anaemia in the third trimester of pregnancy. Those who had mild anaemia at baseline had a probability of 18.8% and 16.5% to develop mild or moderate to severe anaemia later, while the exact probabilities for those with moderate to severe anaemia at baseline were 69.6% and 77.7% (**Figure 2**).

## DISCUSSION

Using data from a community-based pregnancy cohort in a rural district of Bangladesh, in which Hb levels were measured at two time points during pregnancy: at 8–19 weeks (baseline) and at 24–36 weeks of gestation (follow-up), we documented a very high burden of anaemia. About 38% of women were anaemic at baseline, with 24% having mild anaemia and 14% having moderate to severe anaemia, while 79% were anaemic at follow-up, with 30% having mild anaemia and 49% having moderate to severe anaemia. Most women with no anaemia at baseline became anaemic by the third trimester, and few women with anaemia at baseline recovered over the pregnancy. About 6% of the women with no anaemia and about 16% with mild anaemia at baseline developed moderate to severe anaemia at follow-up. About 80% of the women with moderate to severe anaemia at baseline had no change in their condition at follow-up. Several factors, including regular iron supplementation, drinking tube-well water, and consuming meat at least twice per week were associated with reduced risk of developing moderate to severe anaemia.

The prevalence of anaemia in early pregnancy (38%) observed here is similar to that found in studies in Ethiopia (36.6%) [25], in metropolitan Bangladesh (37%) [7], and in Indonesia (40.7%) [26]. The rate of anaemia we found was higher than that observed in Sri Lanka (14.4%) and India (20%) [27,28]. However, the burden of anaemia in our sample was lower than the one reported in an earlier cross-sectional study conducted in Gode town, Eastern Ethiopia (56.8%) [1] and Punjab, Pakistan (65.4%) [29]. The prevalence of anaemia in our population in the third trimester of pregnancy was very high (79%). Other studies conducted in Tanzania (80.8%) and Pakistan (75%) also identified a high anaemia prevalence later in pregnancy [2,30].

We observed that overweight/obese women had a lower risk (RRR = 0.57; 95% Cl = 0.37–0.89) of developing moderate to severe anaemia compared to women of normal weight, which is consistent with other research [31–33]. A Chinese study found that women who are overweight or obese consume more iron than those who have a lower BMI [34]. Although overweight women may appear to have adequate nutritional reserves, excess body weight is often associated with chronic inflammation, which increases hepcidin levels and can impair iron absorption and utilisation. This inflammation can also elevate ferritin concentrations, potentially masking iron deficiency and complicating anaemia assessment [35,36]. We observed a higher risk of anaemia in underweight women, although this association was not statistically significant. However, several other studies documented that underweight women had a higher risk of developing anaemia, indicating the need for policy attention to prioritise the inclusion of iron-folate or multiple micronutrient supplements in maternal-child health programmes [11,37,38].

Iron supplementation modestly reduced the risk of moderate to severe anaemia (RRR = 0.75; 95% CI = 0.60–0.93). Iron deficiency is the most common cause of anaemia, often due to insufficient iron intake, poor iron absorption, or excessive blood loss [39]. Other common causes of anaemia are vitamin B12 or folate deficiency. Pregnancy increases the body’s need for iron, folate, and other micronutrients, which, if unmet, can lead to anaemia, particularly iron deficiency anaemia, which is the most common nutritional deficiency globally, affecting over 1.2 billion people, especially women of reproductive age, pregnant women, and young children [39]. This condition is managed through oral iron supplementation as the first-line treatment due to its affordability and effectiveness. Studies have shown that oral iron supplementation can correct anaemia within 6–8 weeks and replenish iron stores over 3–6 months. However, it often causes gastrointestinal side effects like nausea, constipation, and dark stools, which can affect adherence. Taking iron with food may reduce these side effects, though this may slightly reduce absorption. Using formulations like slow-release or liposomal iron can also serve this purpose. Recent evidence suggests that alternate-day dosing may improve absorption and reduce gastrointestinal side effects compared to daily supplementation [40]. Intravenous iron, meanwhile, is used for individuals who cannot tolerate oral iron and have severe anaemia or malabsorption conditions, as it provides faster correction of iron stores, but carries a risk of allergic reactions [41].

We observed that drinking tube well water reduced the risk of anaemia. This relationship is well-studied in Bangladesh and many other settings [42,43]. Bangladesh relies on tube wells as the primary source of drinking water, with many containing iron-rich groundwater, which can help reduce iron deficiency-related anaemia. Merrill and colleagues [44] found that the consumption of iron-rich tube well water in Bangladesh was associated with lower anaemia prevalence among children and pregnant women, particularly in regions with groundwater iron concentrations above 1 mg/L, indicating it as a natural supplement in iron-deficient populations. While tubewell water in Bangladesh can alleviate the burden of anaemia, this benefit is offset by the harmful effects of arsenic contamination in certain areas of Bangladesh. Effective interventions must account for both factors to address anaemia comprehensively [42].

The risk of moderate to severe anaemia was lower among women who reported consuming meat at least twice a week (RRR = 1.27; 95% CI = 1.01–1.60). However, only two out of five women consumed meat at least twice a week. Consuming iron-rich foods such as meat plays a significant role in managing and preventing anaemia, particularly iron-deficiency anaemia. Meat and fish are rich sources of heme iron, which the human body absorbs more easily than the non-heme iron found in plant-based foods like beans, lentils, and spinach. Incorporating iron-rich foods like meat into the diet could significantly improve iron levels and support the treatment of iron-deficiency anaemia [45,46].

Iron deficiency anaemia is the most common nutritional deficiency worldwide, with widespread effects on health, development, and quality of life. It leads to diminished oxygen delivery to tissues, causing chronic fatigue, weakness, and reduced physical performance, particularly in physically active individuals [47,48]. Adults with this condition may experience poorer work performance, leading to economic losses on both individual and societal levels, and an increased need for medical treatment and management of complications increases the financial burden. Iron is crucial for immune cell function; deficiency can impair immunity, increasing susceptibility to infections [49]. Iron deficiency anaemia affects brain function, particularly in children, and deficiency leads to developmental delays and reduced cognitive performance. Furthermore, it is associated with preterm delivery, low birth weight, and perinatal mortality in pregnant women. Severe anaemia increases the risk of postpartum haemorrhage and maternal mortality [50].

We found no significant association between maternal depressive symptoms (PHQ-9 ≥10) and anaemia in late pregnancy. However, maternal depression may influence anaemia risk by affecting dietary behaviours and adherence to iron-folic acid supplementation, as suggested by studies linking antenatal depression to poorer micronutrient intake [51,52]. Additionally, evidence has indicated a bidirectional relationship between iron deficiency and depression during pregnancy, underscoring the need for further research, despite our null findings [53]

This study has several limitations. First, we conducted it in a rural area of Bangladesh, which limits the generalisability of its findings. We did not measure biochemical markers of iron deficiency (*e.g.* serum ferritin, transferrin saturation), hindering us from distinguishing iron deficiency anaemia from other aetiologies. We also could not account for several significant risk factors, including interpregnancy interval, parasitic infections (*e.g.* hookworm), and detailed dietary diversity measures. We assessed dietary intake using a seven-day recall, which is subject to recall bias. The same limitation applies to the self-reported iron supplementation data collected by CHWs. Furthermore, we excluded 560 women (18.7%) with missing Hb data, which may have influenced both prevalence estimates and risk factor associations. We could not capture detailed information on iron supplementation, including dosage, timing of initiation, duration, or adherence. Finally, anaemia prevalence estimates should be interpreted in the context of gestational age-related hemodynamic changes, which peak in the second trimester, potentially lowering Hb concentrations at 24–36 weeks [54]. The strengths of our study, however, lie in the population-based sampling and its cohort design.

## CONCLUSIONS

We observed a high rate of anaemia among pregnant women in a rural area in Sylhet, Bangladesh, with both the prevalence and severity increasing as gestation progressed. These findings underscore the urgent need for improving nutrition education, expanding access to and adherence with iron supplementation, and enhancing antenatal care, alongside other interventions that could improve maternal and child health outcomes. Future research should also examine system-level and sociocultural determinants of anaemia to better inform context-specific and sustainable intervention strategies.
